# HPO iron chelator, CP655, causes the G1/S phase cell cycle block via p21 upregulation

**DOI:** 10.1002/iid3.342

**Published:** 2020-08-31

**Authors:** Damini Tewari, Katie Lloyd‐Jones, Robert C. Hider, Helen Collins

**Affiliations:** ^1^ Department of Immunobiology School of Immunology and Microbial Sciences, Faculty of Life Sciences and Medicine, King's College London, New Hunts House London United Kingdom; ^2^ Institute of Pharmaceutical Science King's College London London United Kingdom; ^3^ Present address: Damini Tewari, Cambridge Healthcare Research 109‐123 Clifton St London United Kingdom; ^4^ Present address: Katie Lloyd‐Jones, Department of Biological Sciences, Royal Holloway, Centre for Biomedical Sciences University of London Egham Surrey United Kingdom

**Keywords:** cell cycle, CP655, HPO chelators, iron chelation, iron metabolism, p21, T cell proliferation

## Abstract

Iron is known not only for its importance in cellular and metabolic pathways but also for its role in causing cellular toxicities such as production of reactive oxygen species and growth of pathogens. The inability of the human body to physiologically excrete excess iron highlights the need to develop a cheap yet effective iron chelator. This study provides initial evidence of the therapeutic and prophylactic properties of 3‐hydroxypyridin‐4‐one (HPO) chelators in murine collagen‐induced arthritis. To determine whether these chelators would be effective on human cells, we tested a panel of different HPO chelators and identified 7‐diethylamino‐*N*‐((5‐hydroxy‐6‐methyl‐4‐oxo‐1,4‐dihydropyridin‐3‐yl)methyl)‐*N*‐methyl‐2‐oxo‐chromen‐3‐carboxamide (CP655) as the most effective compound targeting human CD4+ T cells. Treatment with CP655 causes significant inhibition of cell proliferation and production of inflammatory cytokines such as interferon‐γ and interleukin‐17. Microarray analysis revealed dysregulation in cell cycle‐related genes following CP655 treatment. This was validated by flow cytometry demonstrating a G1/S phase block caused by CP655. Finally, mechanistic experiments revealed that the chelator may be causing an upregulation of the cell cycle inhibitor protein CDKN1A (p21) as a possible mechanism of action. In conclusion, this study demonstrates that HPO chelators could prove to have therapeutic potential for diseases driven by excessive T cell proliferation.

## Graphical abstract

Novel HPO Iron chelator, CP655, inhibits cell proliferation by blocking the G1/S phase of the cell cycle. This shows potential for its use in treatment of diseases driven by T cell proliferation.

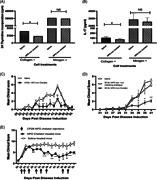

**Abbreviations**
APC: antigen‐presenting cells
CIA: collagen‐Induced Arthritis
CP655: 7‐diethylamino‐*N*‐((5‐hydroxy‐6‐methyl‐4‐oxo‐1,4‐dihydropyridin‐3‐yl)methyl)‐*N*‐methyl‐2‐oxo‐chromen‐3‐carboxamide
DFO: desferrioxamine
DFP: deferiprone
DFX: deferasirox
HPO: 3‐hydroxypyridin‐4‐one
PBMC: peripheral blood mononuclear cells

## 1 INTRODUCTION

The essential role played by iron (Fe) in health and in disease is not disputed.^1^ Many critical life processes including DNA and protein synthesis, cell growth and differentiation and cellular respiration, are dependent on the availability of iron.^2^ However, iron can also have a pathological role in the rapid growth and survival of cancer cells as well as for the proliferation of pathogens such as *Mycobacterium tuberculosis*.^3^, ^4^ It can lead to the production of free oxygen radicals leading to cellular toxicity and has also been implicated in autoimmune disorders such as systemic lupus erythematosus, Goodpasture's Syndrome, multiple sclerosis, and diabetes mellitus I.^5^, ^6^, ^7^, ^8^, ^9^ Furthermore, it has also been implicated in disorders such as atherosclerosis, neuroinflammation, Parkinson's disease, and neuropathic pain.^10^, ^11^, ^12^, ^13^ Hence, maintaining a balance between the availability of iron for essential biological processes and preventing iron misdistribution is of utmost importance for health.

Anemia, is a common complication in several chronic inflammatory disorders such as rheumatoid arthritis (RA), leading to an iron metabolism disorder known as anemia of chronic disease (ACD) or anemia of inflammation.^14^ Under normal physiological conditions, 95% of the body's need for iron is met by iron recycling by macrophages.^15^ However, chronic inflammation leads to alteration of body iron homeostasis and macrophage iron metabolism leading to low levels of circulating iron and high levels of iron accumulated within the cells of the reticuloendothelial system, mainly monocytes and macrophages.^16^

The hypothesis underlying this study is based on a study by Evans et al^17^ which showed that CD14+ monocytes from synovial fluid or synovial membrane from patients with RA were capable of skewing the phenotype of CD4+ T cells, by causing them to produce increased amounts of interleukin‐17 (IL‐17) and interferon‐γ (IFN‐γ) as compared with CD4+ T cells cultured with peripheral blood monocytes. Furthermore, previous studies by Ahmadzadeh et al^18^ and Caccavo et al^19^ have established the presence of excessive iron in synovial fluid and synovial membrane monocytes of RA patients showing signs of ACD, suggesting that the increased iron accumulation might be responsible for inflammation.

On this basis, the aims of this paper were twofold. First, we wanted to identify whether iron chelation could reduce symptoms of chronic inflammation in an animal model of arthritis. Second, to determine whether, and by what mechanism of action, iron chelation reduced inflammatory cytokine production and proliferation of primary human immune cells, namely, CD14+ antigen‐presenting cells (APCs) and CD4+ T cells.

The inability of the human body to physiologically excrete iron highlights the need to develop an effective iron chelator, which can bind and eliminate excess iron from the body.^20^ The past few years have seen an increase in the number of targeted approaches towards the use of iron‐chelating compounds for the treatment of infectious and noninfectious diseases.^21^, ^22^ From being restricted to iron‐overload disorders such as hereditary haemochromatosis, iron chelation as a therapy is now being used in a myriad of inflammatory and infectious disorders.^11^, ^23^, ^24^, ^25^

Until recently, desferrioxamine (DFO) was the only clinically approved iron chelator for use in humans. However, its therapeutic potential is restricted as a result of its limited membrane permeability, short half‐life and toxic side effects.^26^, ^27^, ^28^ Over the last 10 years, the US Food and Drug Authority (FDA) has approved two additional iron chelators, deferasirox (DFX) and deferiprone (DFP) for human use. In addition, several other novel chelators are currently at different stages of clinical trials and will be available for clinical use in the upcoming years. This heightened interest in iron chelation highlights the great potential it offers as a new form of therapy for a range of disorders.^22^, ^29^

The 3‐hydroxypyridin‐4‐one (HPO) family is a bidentate iron chelator group known for its high affinity, selectivity, and specificity to intracellular iron (III).^30^, ^31^ These compounds have lower molecular weights as compared with many of the previously used iron chelators. Additionally, the presence of a neutral charge in both iron‐free and iron‐complexed forms affords them the ability to easily cross cellular membranes and distribute more evenly and freely within the intracellular compartments.^32^ As a result of these properties, the HPO chelators are considered to be one of the main candidates for the development of orally active iron chelators.^31^, ^34^ The HPO chelators used in this study were developed for use in previous studies. The fluorescent probes attached allow these chelators to be used as chemo‐sensors, on interaction with intracellular iron, the fluorescence is quenched.^30^, ^34^ The addition of the fluorescent moiety, while changing the neutral charge on the molecule, did not affect the uptake or intracellular location of the chelator.^30^ Though not used in this particular study, the iron sensing properties of the chelators will be used in future follow‐up studies.

This study focuses on identifying the mechanism of action of novel HPO iron chelators. To this end, the study begins by evaluating a panel of different HPO chelators in collagen‐induced arthritis (CIA) which is a mouse model of RA. As iron accumulation in macrophages has been reported in RA, we examined whether HPO chelators would be successful in reducing inflammation, as a consequence of iron accumulation, in CIA. Once the ability of iron chelation in reducing inflammation was established, the authors further dissected the mechanism of action of the chelators by using human immune cells in an in‐vitro model. Further investigations reveal that from a range of HPO chelators tested, 7‐diethylamino‐*N*‐((5‐hydroxy‐6‐methyl‐4‐oxo‐1,4‐dihydropyridin‐3‐yl)methyl)‐*N*‐methyl‐2‐oxo‐chromen‐3‐carboxamide (CP655) was most effective in inhibiting human CD4+ T cell proliferation by arresting cells in the G1/S phase of the cell cycle.

## 2 MATERIALS AND METHODS

### 2.1 Animals

Male C57BL/6 mice aged 8 to 10 weeks were obtained from Harlan Olac, Bicester, UK. They were housed in specific pathogen‐free conditions and fed ad libitum. All experiments were performed according to UK Home Office guidelines.

### 2.2 CIA mouse model

Type II chicken collagen (Sigma) at 4 mg/mL was emulsified 1:1 with Complete Freund's Adjuvant (Sigma). C57BL/6 mice were injected ip at the base of the tail with two injections of the emulsion at 50 μL per site. The mice were observed daily for signs of ill health and were scored for clinical signs of arthritis three times a week after day 18. The severity of arthritis was graded using an established scoring system^35^: 0 = no arthritis, 1 = 1 inflamed digit, 2 = 2 inflamed digits and/or erythema and mild swelling of the footpad, 3 = >2 digits and footpad inflamed, 4 = all digits and footpad inflamed. An arthritis score for each mouse was calculated by summing up the scores for each paw. If the score was 12 or higher, the mouse was immediately euthanized in accordance to the Home Office guidelines.

### 2.3 Administration of chelator for trials

For the prophylactic trial, mice were injected ip on days −1, 0, and +1 with 200 µL of chelator at a concentration of 250 µM. This was continued biweekly for 2 weeks. For the therapeutic trial, once signs of the disease were observed the mice were injected ip with 200 µL of chelator at a concentration of 250 µM twice a week until the end of the trial.

### 2.4 Human subjects

Blood samples were routinely obtained from healthy volunteers after obtaining ethical approval and consent from each of the donors (REC number: 06/Q0705/20). Blood was used for isolation of peripheral blood mononuclear cells (PBMCs) that were used in all experimental set‐ups.

### 2.5 Isolation of CD4+ T cells and CD14+ monocytes from healthy human PBMC

PBMCs were isolated using standard density gradient centrifugation protocol using lymphocyte separation medium. CD14+ cells were positively selected from isolated PBMCs using magnetically activated cell sorting (MACS) (Miltenyi Biotec). Following this, CD4+ cells were negatively isolated from the CD14− cell fraction using the CD4+ T‐cell isolation kit (Miltenyi Biotec). In all experiments, cells greater than 90% purity, as determined by Fluorescence‐activated cell sorting (FACS) analysis, were used. The cells were counted and diluted to the required concentrations with Rosewell Park Memorial Institute (RPMI) 1640 (Gibco, New Zealand) supplemented with 2 mM l‐glutamine (Lonza, Belgium), 100 U/mL of penicillin + 0.1 mg/mL streptomycin (Lonza, Belgium) and 10% FCS (Gibco, UK).

### 2.6 Culturing CD4+ T cells and CD14+ monocytes

CD4+ T cells and CD14+ monocytes were cultured in a ratio of 2:1 in 1‐mL RPMI 1640 (Gibco, New Zealand) supplemented with 2 mM l‐glutamine (Lonza, Belgium), 100 U/mL of penicillin + 0.1 mg/mL streptomycin (Lonza, Belgium), and 10% FCS (Gibco, UK). Cultures were stimulated with tetanus toxoid (kindly provided by Prof Leonie Taams, KCL) in either the presence or absence of the chelator CP655 (5 μM) or the control CP655OMe (5 μM) for 6 days. Following this, the cells were stimulated with 750 ng/mL ionomycin (Sigma‐Aldrich, Poole, UK) and 50 ng/mL PMA (Sigma‐Aldrich) for 4 hours, supernatants were harvested and used to measure cytokines by enzyme‐linked immunosorbent assay (ELISA). Cells were used to measure proliferation by ^3^H‐thymidine incorporation as described below.

To identify the target cell for the chelator, CD14+ cells and CD4+ cells were incubated separately overnight either with or without CP655/CP655OMe. The following day, they were washed with RPMI (supplemented as above) to remove the chelator and the resulting cells were cocultured in different combinations with tetanus toxoid and further incubated for 6 days. Following this, cells were stimulated with 750 ng/mL ionomycin and 50 ng/mL PMA for 4 hours after which supernatants were harvested and cytokines measured by ELISA. Cells were used to measure proliferation by ^3^H‐thymidine incorporation as described ahead.

### 2.7 Stimulation of CD4+ T‐cell cultures with CD3/CD28

Purified CD4+ T cells were cultured in 1 mL RPMI 1640 (Gibco, New Zealand) supplemented with 2mM l‐glutamine (Lonza, Belgium), 100 U/mL of penicillin + 0.1 mg/mL streptomycin (Lonza, Belgium) and 10% FCS (Gibco, UK). Cells were stimulated with anti‐CD3/CD28 beads (Invitrogen, UK) in a ratio of 1:20 (bead:CD4+ T cells) and treated with or without iron chelator CP655 (5 µM) or control compound CP655OMe (5 µM) for various time points ranging from 18 hours to 6 days posttreatment. In the absence of CD14 + APCs presenting the tetanus toxoid antigen to CD4+ T cells, anti‐CD3/CD28 beads were used to directly stimulate the CD4+ T cells.

### 2.8 ELISA

For murine ELISAs, supernatants from cultured spleen and lymph node cells were collected and stored at −80°C until required or used fresh. IL‐17A and IFN‐γ were measured by ELISA. The ELISA was carried out as described.^35^ Briefly, the capture mAb was coated on 96‐well maxisorp plates for 24 hours at 4°C (Nunc, Denmark). After blocking and washing, the supernatant samples were added. The wells were washed after 24 hours incubation at 4°C and the bound cytokines were labeled with an anticytokine biotinylated mAb directed at different epitopes to the capture antibody. This was visualized by adding streptavidin peroxidase reagent (20 minutes at RT) followed by 3,3,5,5′‐tetramethylbenzidin) (all from Sigma). The reaction was stopped with 2M H_2_SO_4_ and optical densities read at 450 nm using an ELISA plate reader (MRX II; Dynex Technologies Inc. Billinghurst, W. Sussex). The concentration was calculated using linear regression analysis of a standard curve from recombinant cytokine protein.

For human ELISA, cytokines were measured using Human ELISA Ready‐Set‐Go kits (eBioscience, San Diego) for IFN‐γ and IL‐17 following manufacturer's instructions. The standard curve, starting from 500 pg/mL and in serial doubling dilutions, was set up using recombinant human cytokines (eBioscience). Plates were read on the MRX II ELISA plate reader (Dynex Technologies) at 450 nm. The sensitivity of the ELISA kits was 4 pg/mL.

### 2.9 ^3^H‐Thymidine proliferation assay

One hundred microliters of cultured cells were transferred to 96‐well U‐bottomed plates and pulsed with 0.5 μCi/well of ^3^H‐thymidine and incubated for 24 hours at 37°C, 5% CO_2_. The plates were harvested using a Tomtec harvester and the glass fiber filter mats (PerkinElmer, MA) were left to dry for at least 24 hours. The filter mats were treated with liquid scintillation fluid (Scintillant‐Beta plate scint; Wallac) and then counted using a Wallac scintillation counter to measure proliferation.

### 2.10 Cell cycle analysis using flow cytometry

CD4+ T cells, cultured as above with our without the chelator, were lysed with cold absolute ethanol for at least 1 hour at 4°C. Cells were washed with cold PBS before staining with 50 µg/mL propidium iodide (PI) (catalog number: P4864; Sigma‐Aldrich) and 0.1 mg/mL ribonuclease A (Sigma‐Aldrich) in PBS for 20 minutes at 37°C, 5% CO_2_ in a humidified atmosphere. After incubation, the cells were resuspended in the same solution. Routinely, 10 000 cells were acquired immediately by the FACS Canto flow cytometer (Becton Dickinson). The data was acquired as a histogram on a linear scale where the *x*‐axis represented the DNA content and the *y*‐axis showed the relative cell number. Analysis was conducted using the FlowJo software and the histogram was divided into G_0_/G_1_, S, and G_2_/M phase based on the amount of DNA content. Gating strategy is shown in Figure S4.

### 2.11 Western blot analysis

Whole‐cell lysates were prepared using sodium dodecyl sulfate buffer. Protein extracts were separated on 8% to 16% precast Tris‐glycine protein gels (NuSep). To determine the molecular weight, Precision Plus Dual‐Color protein standard (Bio‐Rad, UK) was run along with the samples. The protein samples were transferred on to a polyvinylidene fluoride membrane (GE Healthcare, UK) and used for Western blot analysis. The membranes were probed with antibodies against p21 (12D1; Cell Signalling, UK) used at 1:1000 and Glyceraldehyde 3‐phosphate dehydrogenase (9485; AbCam) used at 1:2000, followed by horseradish peroxidase‐conjugated goat anti‐rabbit (84369; Jackson ImmunoResearch) used at 1:10 000. The membrane was imaged using Bio‐Rad Chemi‐Doc XRS Molecular imager.

### 2.12 Microarray analysis of CD4+ T cells

CD4+ T cells were cultured as described above for 18 hours followed by the extraction of RNA using Qiagen RNeasy Microkit (Qiagen, UK) following the manufacturer's instructions. The quantity and quality of the RNA were assessed using Agilent 2100 Bioanalyser (Agilent Technologies). Affymetrix Gene 1.0 ST array was performed as per Ambion's and Affymetrix's instructions, The Affymetrix GeneChip WT Terminal Labeling Kit (Part no. 900671) was used for the fragmentation and labeling of the complementary DNA. The arrays were stained as per the GeneTitan Setup protocol for Gene 1.1 ST array plates and processed on a GeneTitan Instrument.

### 2.13 Iron chelators

HPO chelators were synthesized as previously described,^33^, ^36^, ^37^, ^38^ dissolved in dimethyl sulfoxide (Sigma‐Aldrich), and stored at a concentration of 50 µM at −20°C. The last step in the process of synthesizing CP655 involves the removal of a protective methyl group by treatment with BCl_3_.^37^ The presence of the protective methyl group renders the compound incapable of binding iron and hence made it suitable for use as the nonchelating control to eliminate off‐target effects. The chelators were diluted to the respective concentrations with RPMI media before use. The structures of the chelators used in this study are provided in Figure S5.

### 2.14 Statistical analysis

For in vitro studies, paired parametric data was compared using the Student paired *t* test. Nonpaired parametric data were compared using unpaired *t* test. Where multiple comparisons were made, an ANOVA was used.

The results have been represented as mean ± standard error of the means for the stated number of replicates. Statistical differences between the data have been calculated using GraphPad Prism 5 software using the appropriate statistical tests as indicated in the respective figure legends.

*P values less than .05 were considered significant, **P < .01 and ***P < .001.

## 3 RESULTS

With iron chelators rapidly being tested and developed for therapeutic use, there is a need now to identify compounds that are not only effective for the intended treatment but are practical to use and free from any adverse side effects.

### 3.1 HPO iron chelators have therapeutic and prophylactic effects in murine CIA

**Figure 1:**
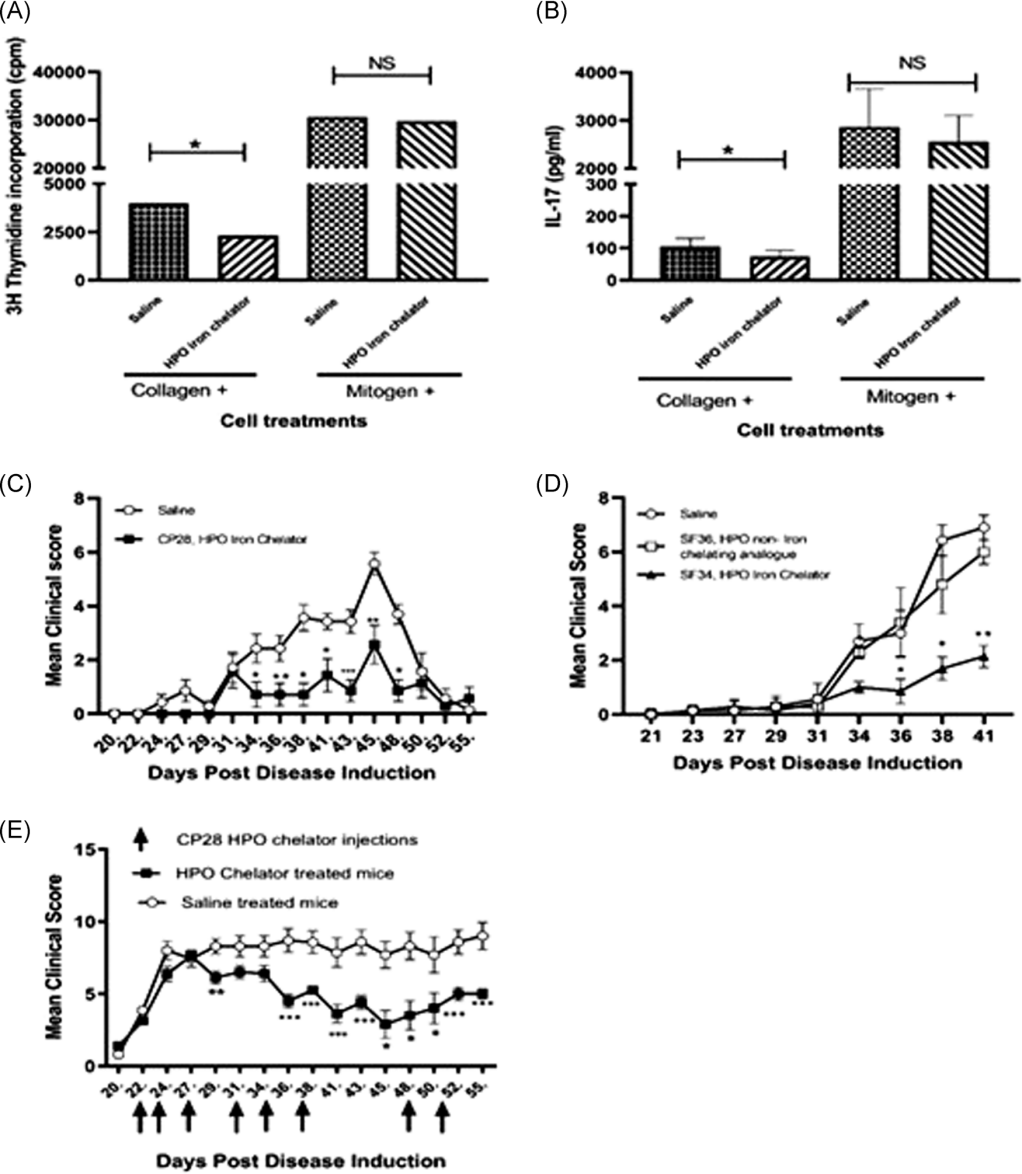
In vitro, prophylactic and therapeutic effect of iron chelation on collagen‐induced arthritis. Mice sensitized to type II collagen were killed 10 days later and spleens and lymph node cell suspensions were restimulated in vitro with type II collagen or Con A, alone or in the presence of 3‐hydroxypyridin‐4‐one (HPO) iron chelator CP28. Proliferation and interleukin‐17 production are shown in (A) and (B), respectively, n = 12. Prophylactic studies were carried out as detailed in the methods section. Briefly, prophylactic intraperitoneal (ip) injections of HPO iron chelator CP28 (C) and SF34 (D) or control compounds, either saline (C) and (D) or noniron‐chelating analog SF36 (D) were given at days −1, 0, and 1, and then twice weekly for 2 weeks. Disease was induced on day 0. Clinical symptoms were scored from day 20, three times a week until the end of the trial (5‐7 mice per treatment group). Therapeutic studies with CP28 were carried out by inducing disease at day 0 (as above) but delaying CP28 ip treatment until mice first demonstrated symptoms of arthritis. Treatment was repeated twice a week until the end of the trial. For (A) and (B), statistical test was a paired *t* test (*P < .05). Error bars denote standard error of the mean (SEM) of duplicate samples. For (C), (D), and (E), data represented as mean ± SEM. n = 7 for each group, *P < .05, **P < .01, and ***P < .001 calculated using Wilcoxon nonparametric test

To test the hypothesis that iron misdistribution is one of the factors responsible for autoimmune and inflammatory disorders such as RA, the murine CIA model was used to test the propensity of HPO iron chelators to improve disease outcomes. Inflammatory cytokines IL‐17 and IFN‐γ and a specific proliferative response to the antigen are hallmark responses in inflammatory diseases such as arthritis. First, we investigated whether the HPO chelators CP28 and SF34 affected inflammatory responses to inflammatory stimulators such as type II collagen. To this end, spleen and lymph nodes were harvested from mice sensitized to type II collagen, cell suspensions made, and cultured with type II collagen with or without HPO iron chelator. The data shown here is for HPO iron chelator CP28 (Figure 1A). The murine proliferation response to type II collagen was significantly lower in the presence of HPO iron chelator CP28, indicating a reduction in inflammatory proliferation to type II collagen (Figure 1A). Mitogenic stimulation in the presence or absence of CP28 induced substantial cell proliferation, indicating that the cells were viable but nonspecific proliferation was unaffected by the presence of CP28. Similarly, type II collagen‐induced IL‐17 production was also significantly reduced in the presence of iron chelator CP28; whereas mitogen‐induced IL‐17 production was unaffected (Figure 1B). Similar results were found using the alternative HPO iron chelator SF34 (data not shown). These results suggest that CP28 HPO iron chelators are able to reduce murine inflammatory responses ex vivo. Next, to see if the same effects were observed in vivo, we used an established mouse model of arthritis induced by type II collagen emulsified in CFA injected intradermally. Initial toxicity studies using a panel of the HPO iron chelators demonstrated that no signs of ill health or weight loss were observed in mice following intraperitoneal (ip) administration of the chelators (data not shown). To investigate the prophylactic effects of the HPO iron chelators, control mice and chelator‐treated mice were injected with either saline or chelator according to the protocol detailed in the methods section. Saline‐treated control mice developed symptoms of arthritis (swelling in paws) from 21 days post disease induction, reaching a peak clinical score around 45 days which then started to resolve (Figure 1C). In contrast, the CP28‐treated mice demonstrated significantly lower clinical scores throughout the time course of the experiment. In a similar experiment, the prophylactic effect of a second HPO iron chelator SF34 was investigated. Again, compared with saline‐treated controls, mice treated with SF34 demonstrated significantly reduced clinical scores (Figure 1D). In contrast, the noniron‐chelating HPO analog, SF36 did not impact the clinical scores, supporting the importance of the iron‐chelating aspect of SF34 in inhibiting the induction of arthritis in this model. To investigate the potential therapeutic effects of HPO chelators, treatment with CP28 was delayed until mice first demonstrated symptoms of arthritis, which occurred at approximately day 22 post disease induction (Figure 1E). Repeated treatment with CP28 was able to significantly reduce the severity of disease compared with mice treated with saline. Taken together, these data indicate that HPO iron chelators, when given as prophylactic or therapeutic treatment, have the capacity to significantly reduce the clinical signs of arthritis so improving disease outcome.

### 3.2 Identification of CP655 as the most effective HPO chelator

To identify a suitable candidate, we evaluated a panel of HPO chelators of varying affinity for intracellular iron. As use of iron chelators such as DFP, DFO, and DFX to treat inflammatory disorders has been previously established,^39^, ^40^ and following our own observations on murine cells, we assessed the effect of the HPO chelators on cell proliferation and the production of the inflammatory cytokines, IFN‐γ and IL‐17 from co‐cultures of CD4+ T cells and CD14+ monocytes, stimulated with tetanus toxoid.

**Figure 2:**
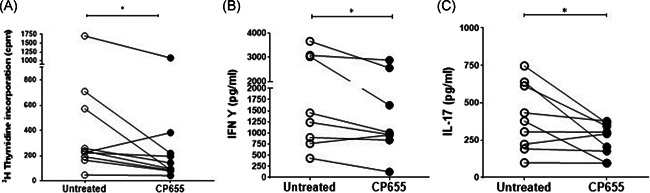
CP655 (7‐diethylamino‐*N*‐((5‐hydroxy‐6‐methyl‐4‐oxo‐1,4‐dihydropyridin‐3‐yl)methyl)‐*N*‐methyl‐2‐oxo‐chromen‐3‐carboxamide) inhibits T cell proliferation and cytokine production. CD4+ (T cells) and CD14+ (monocytes) cells were isolated from fresh blood of healthy donors. Cells were mixed in a ratio of 2:1 (T cells: Monocytes), stimulated with tetanus toxoid and treated with or without CP655 (5 µM). After 6 days of incubation, cells were removed for measuring proliferation and the remaining cells were stimulated with 750 ng/mL Ionomycin and 50 ng/mL PMA for 4 hours. Proliferation was measured by incorporation of ^3^H‐thymidine (A). Cytokines were measured by enzyme‐linked immunosorbent assay (B, C). Each symbol represents an individual donor n = 8 to 10. *P < .05 calculated using Wilcoxon nonparametric matched‐pairs test

HPO chelators showed differing effects on proliferation and cytokine production (Figure S1). CP655 was noted to be the most effective chelator in significantly reducing proliferation, IFN‐γ and IL‐17 production in the cultures as compared with the untreated controls (Figure 2). In light of the published data demonstrating the role of iron chelation in reducing cell growth and division, cell proliferation was used as the main measure in the following experiments.

**Figure 3:**
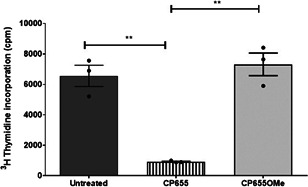
CP655 (7‐diethylamino‐*N*‐((5‐hydroxy‐6‐methyl‐4‐oxo‐1,4‐dihydropyridin‐3‐yl)methyl)‐*N*‐methyl‐2‐oxo‐chromen‐3‐carboxamide) effect on CD4+ T cell proliferation is iron‐dependent. CD4+ (T cells) and CD14+ (monocytes) cells were isolated from fresh blood of healthy donors. Cells were mixed in a ratio of 2:1 (T cells:monocytes), stimulated with tetanus toxoid, and treated with or without CP655 (5 μM) or the control compound CP655Ome (5 μM). Proliferation was measured by incorporation of ^3^H‐thymidine. Data represented as mean + standard error of the mean from n = 3 individual donors. **P < .01 between cultures with absence and presence of CP655 or CP655OMe calculated using the unpaired *t* test

We confirmed that the inhibitory effects observed following CP655 treatment resulted from its ability to chelate intracellular iron rather than an off‐target effect. To this end, a compound structurally identical to CP655, but with its iron‐chelating moiety masked by a methyl group was synthesized (CP655OMe). In a coculture of tetanus toxoid stimulated CD4+ T cells and CD14+ monocytes, treatment with CP655 significantly reduced cellular proliferation, whereas treatment with the nonchelating compound, CP655OMe, had no effect on the proliferation of the cells (Figure 3). Furthermore, to confirm that reduction in cell proliferation was not a result of toxicity, PI staining was used to measure cell viability after CP655 and CP655OMe treatment. Untreated cells showed an average of 1.0% ± 0.6% cell death after culture, whereas treatment with CP655 and CP655OMe resulted in an average of 1.5% ± 0.8% and 2% ± 0.5% cell death, respectively.

### 3.3 CD4+ T cells are the target cell for CP655‐mediated iron chelation

**Figure 4:**
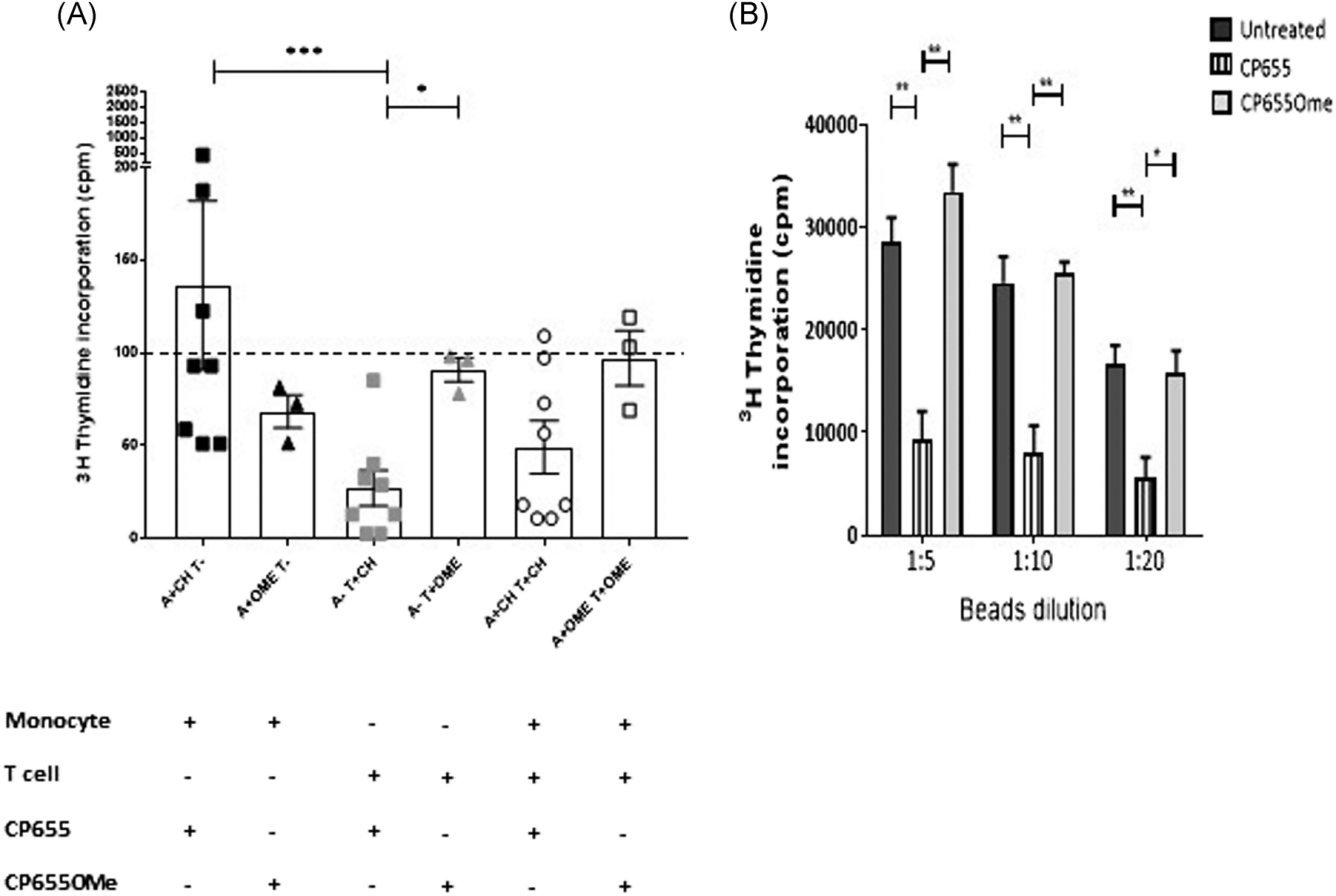
CP655 (7‐diethylamino‐*N*‐((5‐hydroxy‐6‐methyl‐4‐oxo‐1,4‐dihydropyridin‐3‐yl)methyl)‐*N*‐methyl‐2‐oxo‐chromen‐3‐carboxamide) targets CD4+ T cells. A, CD4+ (T cells), and CD14+ (monocytes) cells were isolated from blood of healthy donors. Cells were incubated separately with or without CP655 or CP655OMe for 24 hours. The cells were washed and mixed in different combinations and stimulated with tetanus toxoid. Proliferation was measured by incorporation of ^3^H‐thymidine. Each symbol represents an individual donor n = 3 to 8. Dotted line represents control cultures where both CD4+ and CD14+ cells were untreated. Results expressed as a percentage of the control. *P < .05 calculated using the Mann‐Whitney test. B, CD4+ cells were isolated from fresh blood of healthy donors. Cells were stimulated with anti‐CD3/CD28 beads at the indicated ratios in the presence of 5 μM CP655 or 5 μM CP655OMe for 24 hours. Proliferation was measured by incorporation of ^3^H‐thymidine. Results represented as mean + standard error of the mean from n = 5 individual donors. **P < .01 and *P < .05 calculated using the Holm‐Sidak *t* test

To elucidate which cells in the monocyte/CD4+ T cell coculture were affected by CP655, CD4+ T cells, and CD14+ monocytes were separately cultured with either media, CP655 or the control CP655OMe, and then washed before being cocultured and stimulated with tetanus toxoid as previously described. The results demonstrated that CP655 reduced proliferation only when CD4+ T cells were pretreated, as opposed to when CD14+ cells were pretreated with the compound. Treatment of both CD14+ and CD4+ T cells was also found to reduce proliferation but to a lesser degree than when only CD4+ T cells were pretreated (Figure 4A). Treatment of either CD4+ T cells or CD14+ monocytes with CP655OMe did not induce any effect on cellular proliferation. These experiments demonstrated that CP655 interfered with the proliferation of CD4+ T cells but had no effect on CD14+ cells.

CD14+ APCs presenting the tetanus toxoid antigen were required for stimulating the CD4+ T cells in the previous experiment, which in turn caused inflammatory cytokine production and cell proliferation. To further confirm that CP655 had an impact directly on CD4+ T cells, we replaced CD14+ APCs with anti‐CD3/CD28 beads. Treatment with CP655 resulted in a 75% inhibition of proliferation at each of the bead ratios, whereas CP655OMe did not show any effect (Figure 4B). In addition, these results demonstrated that the chelator retained its inhibitory effects at higher levels of stimulation. It was decided to use the lowest concentration of the anti‐CD3/CD28 beads for stimulating CD4+ T cells (that is 1:20 bead:CD4+ T cells ratio) in subsequent studies.

### 3.4 CP655 inhibits proliferation by interfering with cell growth and developmental pathways

**Figure 5:**
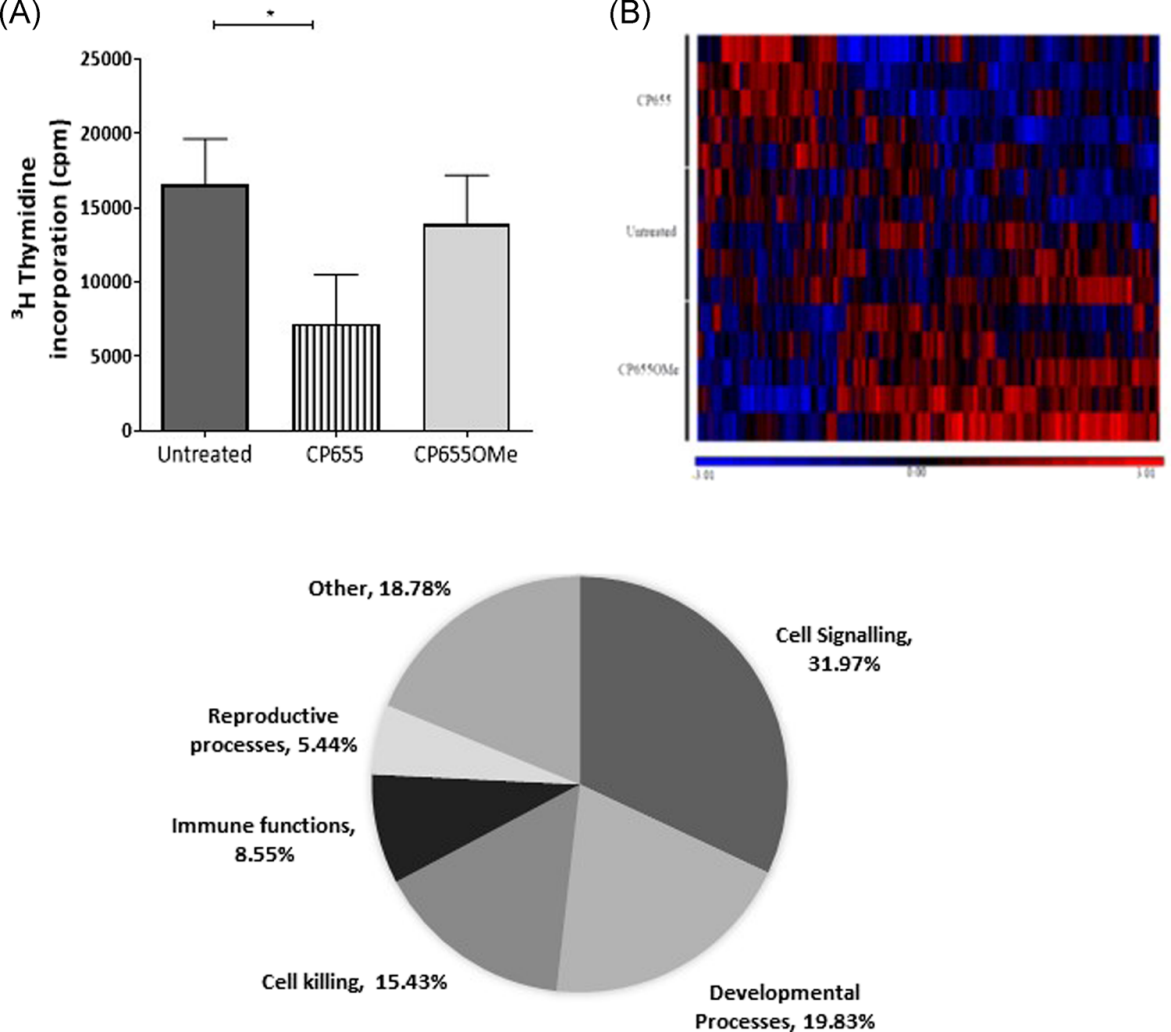
Microarray analysis of CD4+ cells treated with CP655 (7‐diethylamino‐*N*‐((5‐hydroxy‐6‐methyl‐4‐oxo‐1,4‐dihydropyridin‐3‐yl)methyl)‐*N*‐methyl‐2‐oxo‐chromen‐3‐carboxamide) vs CP655OMe. Cells from five individual donors were either left unstimulated or stimulated with anti‐CD3/CD28 beads and left untreated or treated with either CP655 or CP655OMe for 18 hours. Extracted messenger RNA was used for microarray hybridization and analysis. A, Proliferation of CD4+ T‐cell samples used for microarray. CD4+ T cells were isolated from peripheral blood mononuclear cells of healthy donors. Cells were stimulated with either anti‐CD3/CD28 beads (1:20) and left untreated or treated with either CP655 (5 µM) or the control CP655OMe (5 µM) for 18 hours. Proliferation was measured by ^3^H‐thymidine incorporation. *P < .05 calculated using the paired *t* test. Data represented as mean ± standard error of the mean from n = 5 individual donors. B, Heat map prepared by GeneSpring Software shows hierarchical clustering using Pearson correlation. Each row represents results from an individual microarray chip (n = 15) showing three treatment conditions for each of the five donors. Each column represents an individual gene. Genes have been clustered according to similarities in patterns of expression as shown by the horizontal axis, as well as, by treatment condition in the vertical axis. Treatment conditions are color‐coded with red showing CP655‐treated cells, Orange bar showing CP655OMe‐treated and yellow showing untreated cells. Difference in expression level can be distinguished on the heat map based on color with high expression genes in red, intermediate expression in black and low expression genes in blue. C, Pi‐chart illustrating the most differentially modulated cellular pathways between CP655 and CP655OMe treatments. GO‐ontology software was used to determine pathways to which most of the differentially modulated genes belonged

To determine which cellular pathways were impacted by CP655 treatment of CD4+ T cells, a microarray was conducted to provide a snapshot of the altered cellular mechanisms. Before conducting the microarray, the kinetics of CP655 treatment were established by observing the inhibition of proliferation over a 4‐ to 48‐hour period, posttreatment. The results revealed that while a trend in reduced proliferation of CD4+ T cells was observed from as early as 4 hours posttreatment, it was from 18 hours onwards that a highly significant reduction in proliferation of CD4+ T cells was observed (Figure S2). Hence, 18 hours was selected as the time point at which microarray was conducted. In addition to determining the appropriate time point, CD4+ T cell proliferation was measured in the samples to confirm that CP655 had inhibited cellular proliferation, before the samples were used for microarray analysis. Figure 5A shows the proliferation results from the five donors selected for microarray analysis, as before, CP655 significantly reduced CD4+ T cell proliferation as compared with untreated cells and CP655OMe‐treated control cells.

The results obtained from the whole‐genome microarray produced a list of 33 297 differentially expressed genes in the first instance. The list of genes was generated based on the expression of differentially modulated genes following CP655 and CP655OMe treatment, with an unadjusted *P* < .05 in each of the five donors tested. A heatmap was generated by unsupervised hierarchical clustering of data. The clustering used here was Agglomerative, using Euclidean dissimilarity and average linkage (Figure 5B). Each colored column on the heat map represents an individual gene that was significantly modulated when comparing CP655‐ and CP655OMe‐treated samples.

The list of genes that was generated after the first analysis of data revealed many functionally unknown genes. Therefore, to uncover specific signaling pathways that may have been affected due to the treatment, Partek and GeneGo software were used to conduct a pathway analysis of the data generated. The initial results from the pathway analysis showed that the pathway most affected by the treatment with CP655 as compared with CP655OMe was the cell signaling pathway, comprising 32% of the total pathways affected, followed by pathway controlling cell developmental processes and cell killing, respectively (Figure 5C).

To further extend the analysis, the list of genes generated by the differential microarray analysis was manually cross‐referenced with the literature with the aim of identifying important genes that have been previously associated with iron chelation, iron regulation, and cell signaling and cell growth. This led to the identification of 19 genes that were significantly modulated by CP655 treatment (Table 1). It was evident that 53% (10 of 19) of the genes were related to modulation of the cell cycle, with several cell cycle proteins, cyclins, and cyclin‐dependent kinase (cdk) inhibitors amongst the genes significantly modulated by CP655 treatment. Although the genes identified in this list showed a relatively modest foldchange after treatment with CP655, the changes were highly significant.

**Table 1:** Significantly modulated genes following treatment with CP655

Gene	Foldchange	Function
CDC6	−1.2298	Initiation of DNA replication and complete replication of DNA before mitosis. Interacts with 4, iron 4, sulfur clusters.^41^
DLGAP5	−1.1417	Knockdown of DLGAP5 inhibits cell proliferation by increasing p53.^42^
CLSPN	−1.1209	Required for cell cycle progression and movement through S phase.^43^
GINS4	−1.0741	Initiation and progression of DNA replication, by moving ahead of the replication fork and unwinding double‐stranded DNA.^44^
IREB2	−1.0712	Cellular iron homeostasis. Binds to Iron‐Responsive elements in the 5′ and 3′ UTR of Ferritin and Transferrin, respectively.^45^
CDC25A	−1.0563	Transition from G1 to the S phase of the cell cycle by activating cell‐dependent kinase, CDC2 and other cell cycle proteins.^46^
ANAPC1	−1.0557	E3‐ubiquitin ligase that controls the progression of cells through G1 phase of cell cycle.^47^
NAA35	−1.0547	Negative regulator of apoptotic processes. Inhibition causes p53‐dependent apoptosis.^48^
SMAD1	−1.0393	Regulates and mediates several signaling pathways of cell growth, apoptosis, and immune responses.^49^
MIR320A	−1.0314	Inhibits cellular proliferation.^50^
RRM1	−1.0277	Synthesis of deoxyribonucleotides from ribonucleotides, necessary for synthesis of DNA.^51^
GINS3	−1.0245	Initiation of DNA replication and progression of DNA replication fork.^44^
CACNB4	1.15305	Role in Calcium channel functioning by controlling G protein inhibition.^52^
CDKN1A	1.15253	Blocks cellular proliferation by inhibiting cyclin‐dependent kinase activity. Can selectively bind to metal ions.^53^
GADD45G	1.1182	Regulation of growth and differentiation via positive control of Apoptotic pathways.^54^
TGFB1	1.06621	Mature protein regulates cellular proliferation, adhesion, migration, and differentiation, upregulated in several tumor cells.^55^
CDCA4	1.03991	Regulation of cell division and cell proliferation via E2F/RB pathway.^56^
MIR503	1.03019	Increased expression associated with inhibition of proliferation and metastasis in Hepatocellular Carcinoma.^51^
MIR99A	1.00657	Shown to act as a tumor suppressor by inhibiting proliferation in bladder cancer.^57^

*Note:* Significantly modulated genes from the microarray analysis were cross‐referenced to the literature to look for connections to cell growth and signaling, iron chelation, and iron regulation.

Abbreviations: CP655, 7‐diethylamino‐*N*‐((5‐hydroxy‐6‐methyl‐4‐oxo‐1,4‐dihydropyridin‐3‐yl)methyl)‐*N*‐methyl‐2‐oxo‐chromen‐3‐carboxamide; UTR, untranslated region.

On the basis of the indications provided by this microarray data, it was decided to validate the above observations by using flow cytometry to analyze the cell cycle of CD4+ T cells subsequent to various treatments.

### 3.5 CP655 causes the G1/S phase arrest of CD4+ T cells

**Figure 6:**
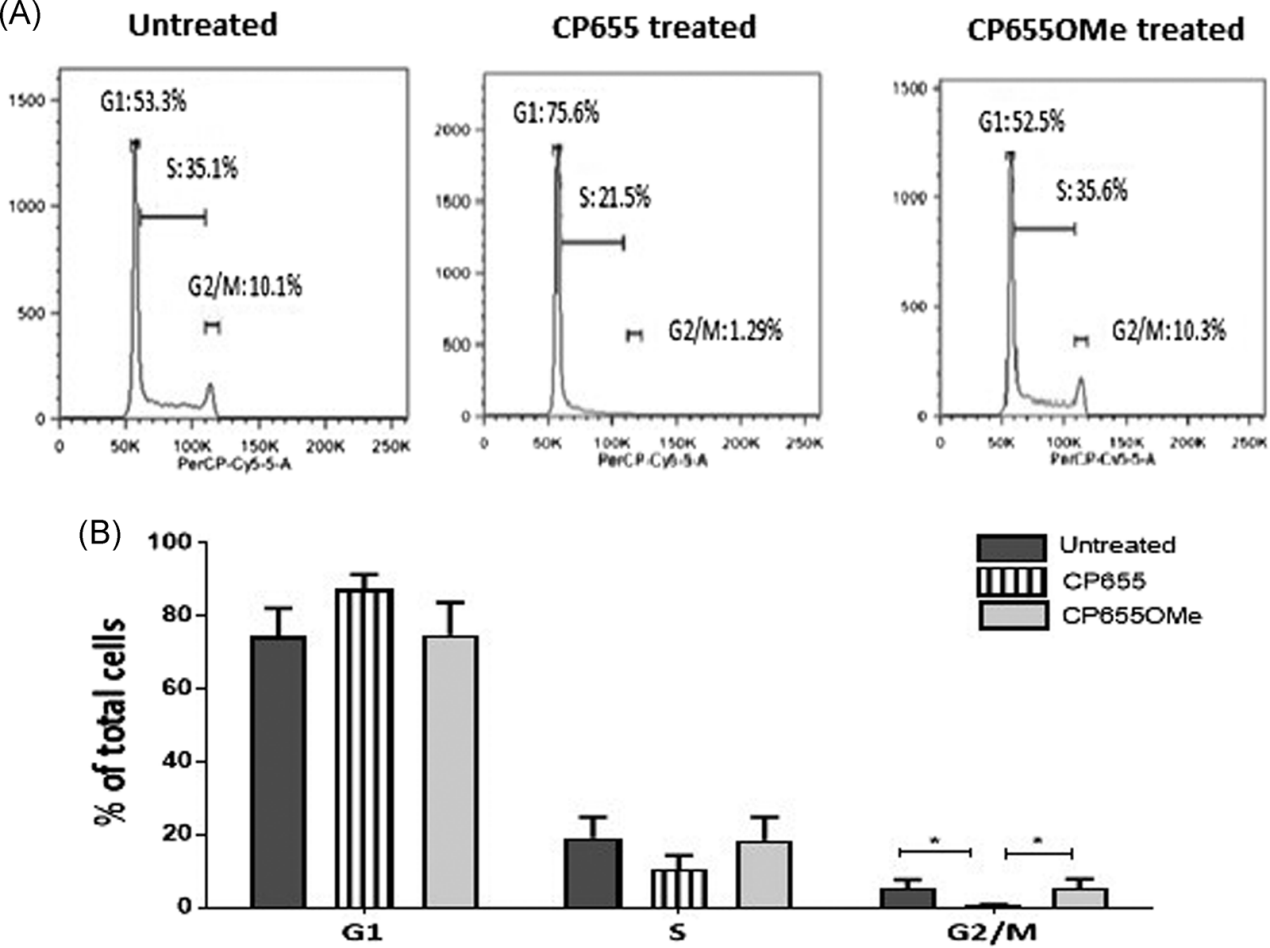
Cell cycle arrest of CD4+ T cells following CP655 (7‐diethylamino‐*N*‐((5‐hydroxy‐6‐methyl‐4‐oxo‐1,4‐dihydropyridin‐3‐yl)methyl)‐*N*‐methyl‐2‐oxo‐chromen‐3‐carboxamide) treatment. CD4+ T cells were isolated from fresh peripheral blood mononuclear cells of healthy donors. Cells were either left unstimulated or stimulated with 1:5 bead:cells ratio of anti‐CD3/CD28 beads in the presence or absence of either CP655 (5 µM) or CP655OMe (5 µM) for 48 hours. Cells were lysed with cold 100% ethanol and stained with propidium iodide and ribonuclease A, before analysis by flow cytometry. A, Representative Fluorescence‐activated cell sorting results from one experiment. B, Cumulative data showing cell cycle arrest of CD4+ T cells from n = 4 donor. Results shown as mean ± standard error of the means. *P < .05 calculated using the paired *t* test

Flow cytometry was adopted to observe the profile of cycling CD4+ T cells. CD4+ T cells were cultured as previously and treated with either CP655 or CP655OMe. After 48 hours of culture, the untreated, as well as the CP655OMe‐treated cultures, had 75% cells in the G1 phase, whereas CP655‐treated cultures showed a consistent increase in the number of cells in the G1 phase (on average, 88% of the cells). Subsequently, CP655‐treated cultures led to a reduction in the percentage of cells entering the S phase (11% vs 19%), and an ensuing significant reduction in the percentage of cells present in the G2/M phase of the cell cycle (0.5% vs 5%) as compared with untreated cells. The control compound CP655OMe consistently showed a profile similar to that of the untreated cells (Figure 6A,B).

These results corroborated the indications provided by the microarray data, confirming that CP655 treatment was indeed interfering with the normal progression of the cell cycle.

### 3.6 CP655 blocks cell cycle progression via p21 protein upregulation

**Figure 7:**
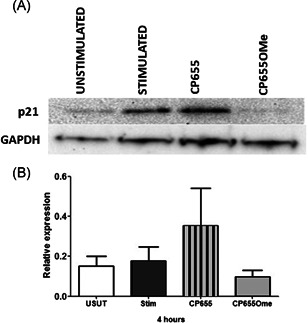
Effect of CP655 (7‐diethylamino‐*N*‐((5‐hydroxy‐6‐methyl‐4‐oxo‐1,4‐dihydropyridin‐3‐yl)methyl)‐*N*‐methyl‐2‐oxo‐chromen‐3‐carboxamide) treatment on p21 protein expression in CD4+ T cells. CD4+ T cells were isolated from peripheral blood mononuclear cells of healthy donors and either left unstimulated (USUT) or stimulated with anti‐CD3/CD28 beads (1:20) in the presence or absence of either CP655 (5 µM) or CP655OMe (5 µM). After 4 hours of culture, p21 expression was analyzed by Western blot analysis. A, Results from one representative experiment. B, Results represented as mean ± standard error of the mean from n = 4 individual donors

Having confirmed the block in the cell cycle following CP655 treatment, experiments were designed to identify the specific proteins causing these changes. Some of the genes from Table 1 were short‐listed for validation by reverse transcription‐polymerase chain reaction (RT‐PCR) and Western blot analysis. Transcriptional analysis was conducted at time points ranging from 4 to 48 hours poststimulation (data not shown). However, no significant changes were visible in any of the genes tested. The only gene transcript that showed a consistent trend towards upregulation following CP655 treatment was CDKN1A (p21). This gene was further analyzed for its protein expression and CP655 was found to upregulate the protein expression of p21 (Figure 7A,B).

Taken together these results suggest that CP655 exerts its antiproliferative effects on CD4+ T cells via its ability to modulate the cell cycle proteins, specifically, the cell cycle inhibitor, p21.

## 4 DISCUSSION AND CONCLUSION

The requirement for iron by metabolically active cells is pivotal, especially for thymocytes and activated T cells. Due to their high rate of metabolism and increased proliferation, their dependence on iron is greater than most other cell types in the human body.^58^ Therefore, whether it is carrying oxygen in hemoglobin, acting as an electron donor or acceptor in various enzymes, producing damaging free hydroxyl radicals or promoting microorganism growth, the impact of iron misdistribution can lead to several immune dysfunctions and even autoimmunity.^8^, ^59^

HPO iron chelators were originally developed to overcome the shortcomings of previously used iron chelators such as DFO.^60^ Past studies have suggested that DFO can cause cellular toxicity leading to apoptosis, cell shrinkage and chromatin fragmentation.^61^ Due to the lower molecular weight of HPO chelators and their ability to cross cell membranes, they diffuse freely within the cell.^32^ This study established the potential of HPO chelators for not only preventing, but also treating, CIA in a murine model, and extends these findings to demonstrate the effects of this class of chelators on human CD4+ T cells.

In agreement with murine studies where HPO chelators decreased both T cell proliferation and inflammatory cytokine production, CP655 was the most effective chelator in reducing human CD4+ T cell proliferation following coculture with CD14+ monocytes, with a concomitant decrease in IFN‐γ and IL‐17 production. This is consistent with other studies where fluorescent iron chelators were used as iron sensors and demonstrated that out of several HPO chelators tested, CP655 demonstrated the maximum sensitivity to intracellular iron.^33^

Our initial hypothesis was that the main target cell for the action of the iron chelators would be APCs such as macrophages (murine) and CD14+ monocytes (human). This was based on previous studies illustrating that under inflammatory conditions, macrophages have been shown to accumulate iron due to changes in expression levels of iron transporting proteins such as divalent metal transporter 1 and ferroportin.^62^, ^63^ Also, inflammatory cytokines such as IL‐6 are known to stimulate hepcidin production, leading to further sequestration of iron by macrophages.^64^ Furthermore, iron‐overloaded macrophages are responsible for the production of reactive oxygen species and inflammatory cytokines, further fueling inflammation.^65^ More recently, Pereira et al^66^ have demonstrated that iron deprivation can reprogram human macrophage metabolism and reduce inflammation in vivo. It was hypothesized, therefore, that chelator treatment would reduce the accumulation of iron in APCs leading to reduced inflammation. However, contrary to expectations, our results indicated that rather than impacting the APCs, HPO chelators demonstrated a more prominent effect on the CD4+ T cells. Previous research has shown that rapidly proliferating lymphocytes up‐regulate transferrin receptors (TfR1) to acquire sufficient iron to fuel their proliferation, and this is found to take place to a greater degree in T cells.^67^, ^68^, ^69^ Microarray analysis of CD4+ T cells treated with CP655 and compared with CD4+ T cells treated with CP655OMe revealed that some of the most significantly modulated genes were those controlling for cell cycle progression as previously suggested by Lewis et al,^70^ Darnell and Richardson^71^ and Deb et al.^72^ Saletta et al^42^ have previously demonstrated that the main pathways that are differentially modulated by iron chelation are those controlling cellular proliferation, cell cycle, and apoptosis.

We confirmed the outcome of the microarray investigation using flow cytometry analysis, demonstrating that CP655‐treated cells are arrested in the G1 phase, with a significant reduction in cells reaching the G2/M phase, when compared with untreated cells. One mechanism by which iron chelators cause an aberration in the cell cycle has been linked to the inhibition of the DNA synthesis enzyme ribonucleotide reductase (RR), which is dependent on iron for its function.^73^, ^74^ Bidentate chelators have been shown to rapidly inhibit RR activity by binding to the iron atoms situated at the active site of the enzyme, as compared with larger hexadentate chelators such as DFO^75^, ^76^, ^77^ (Abeysinghe et al, 1996). The microarray analysis revealed that the larger α subunit of RR, RRM1, was one of the proteins that were significantly modulated (*P* = .02) and downregulated on treatment with CP655.

In accordance with studies indicating the regulation of cell cycle genes by iron chelation, the other genes that were found to be significantly modulated by CP655 treatment included CDC6 (downregulated 1.2‐fold, *P* = .004), ANAPC1 (downregulated 1.05‐fold; *P* < .001), cdk20 (downregulated 1.11‐fold; *P* = .02) and p21 (upregulated 1.15‐fold; NS). Western blot analysis verified the results of the microarray and found that p21 (CDKN1A) protein levels were increased following CP655 treatment, indicating its involvement in the inhibition of the cell cycle. The CDK inhibitor p21 plays a fundamental part in regulating cell cycle progression, as well as senescence and apoptosis. p21 inhibits CDK activity by binding cyclin‐CDK complexes, and thereby induces the G1‐checkpoint after DNA damage.^77^, ^78^ In addition, p21 has also been implicated in G2‐checkpoint maintenance, hence, it plays an important part in various stages of the cell cycle. Other classes of iron chelators, for example, di‐2‐pyridylketone 4,4‐dimethyl‐3‐thiosemicarbazone (Dp44mT) have also been shown to induce the p21 promotor, which reduces proliferation in neoplastic cells.^79^, ^80^

One of the limitations of this study is that some of the changes observed following the microarray analysis were not validated by RT‐PCR. One of the reasons could be that the microarray was only conducted at one time point, hence not providing sufficient information for changes taking place at other time points. Furthermore, there is inherent variability in transcriptional analysis that prevents smaller changes in gene expression from becoming statistically significant. Variability in transcriptional analysis is a common phenomenon^81^, ^82^ since transcription is not a continuous process, but rather a discontinuous one that takes place in “bursts” and “pulses.” Hence differences in the expression levels of lowly and highly expressed genes can be observed even in the absence of any stimulus leading to the observed variability.^83^

Previous studies suggest multiple mechanisms of action for iron chelators including modulation in the level of apoptosis‐related proteins such as Bcl‐2 and Bax^84^ and increase in levels of various caspase proteins.^85^ Reports suggest that alterations in the cell cycle are triggered because of changes to the expression of cyclins and cdks.^86^ Previous studies have shown changes in the level of cyclin‐D1, cyclin‐E, cdk2, cdk4, and reduction of phosphorylated pRB as potential mechanisms of action for various chelators.^87^, ^88^, ^89^

In conclusion, this study reports the properties of a panel of novel HPO chelators and identified CP655 as a potent, chelator able to cause a significant G1/S phase cell cycle arrest of primary CD4+ T cells, as a result of a broad spectrum of transcriptional changes and more specifically by upregulating protein expression of the cell cycle inhibitor, p21.

Results presented here have clearly indicated that HPO chelators can induce anti‐inflammatory effects^32^, ^90^ and this is supported by recent data where^91^ used another HPO chelator, DIBI, and highlighted its superior performance when compared with other chelators in its anti‐inflammatory effects, following sepsis.

## CONFLICT OF INTERESTS

The authors declare that there are no conflict of interests.

## AUTHOR CONTRIBUTIONS

DT performed the in vitro cell cultures, transcriptional analysis of the microarray data, and flow cytometry experiments and analysis. In vivo disease study conducted by KL‐J and HC. HC and DT designed the experiments. RCH contributed towards study design and discussions on the data and results and provided the iron chelators. DT and HC wrote the manuscript.

## Supporting information

Supporting information

Supporting information

Supporting information

Supporting information

Supporting information

## Data Availability

The data that support the findings of this study are available from the corresponding author upon reasonable request.
