# Supplementary figures and images for: HPO iron chelator, CP655, causes the G1/S phase cell cycle block via p21 upregulation
Source: Immun Inflamm Dis. 2020 Aug 31;8(4):568–83. doi: 10.1002/iid3.342 (PMC7654408; doi:10.1002/iid3.342)

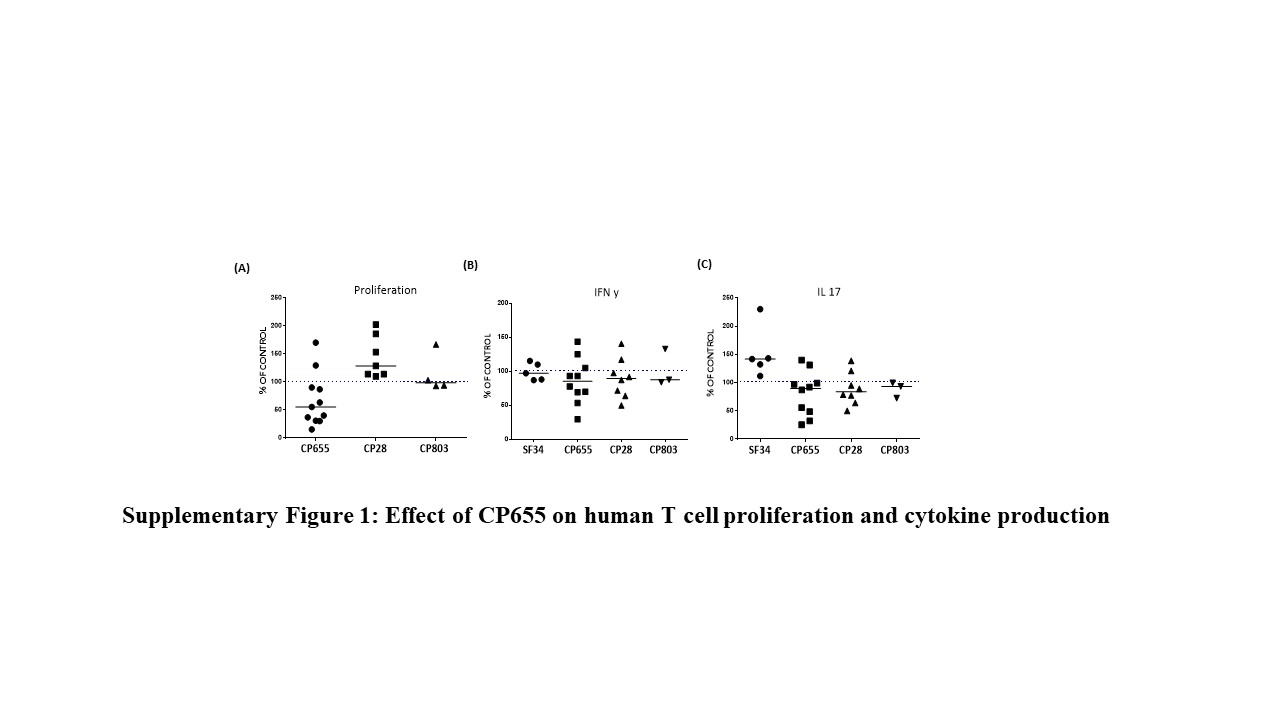

Supplement: Supplementary file 1 — Supporting information [file IID3-8-568-s001.jpg]

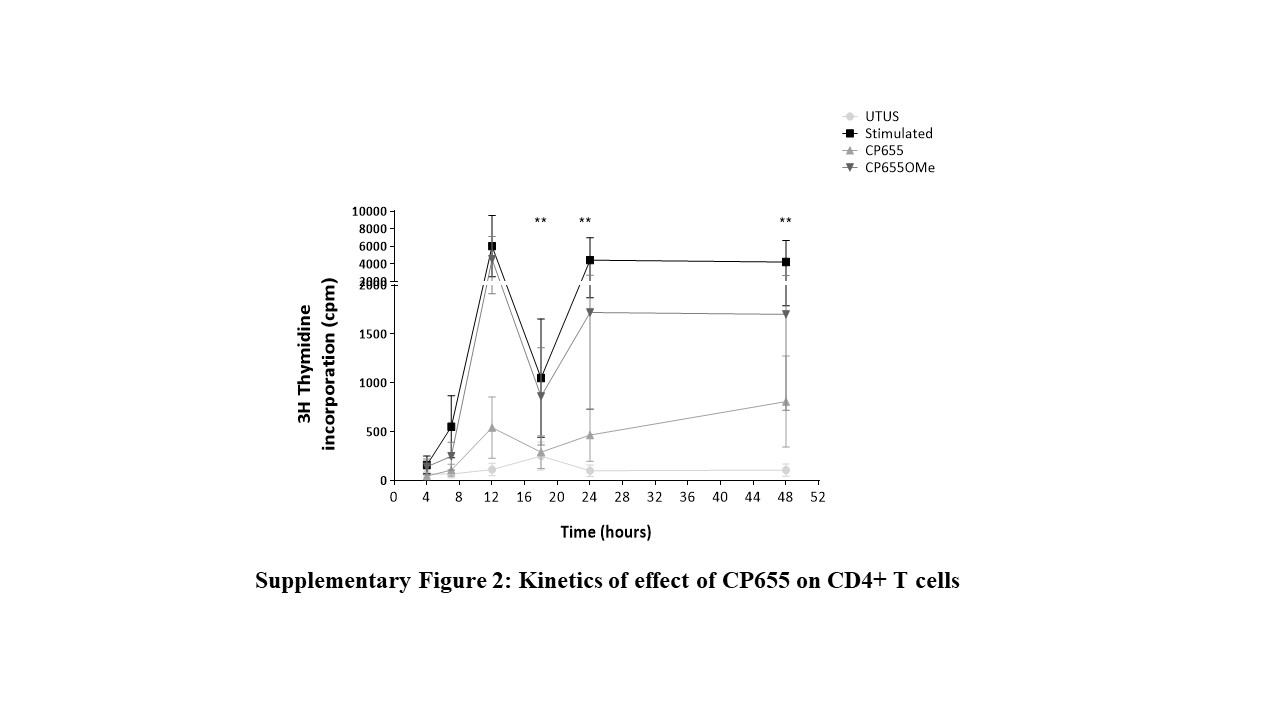

Supplement: Supplementary file 2 — Supporting information [file IID3-8-568-s002.jpg]

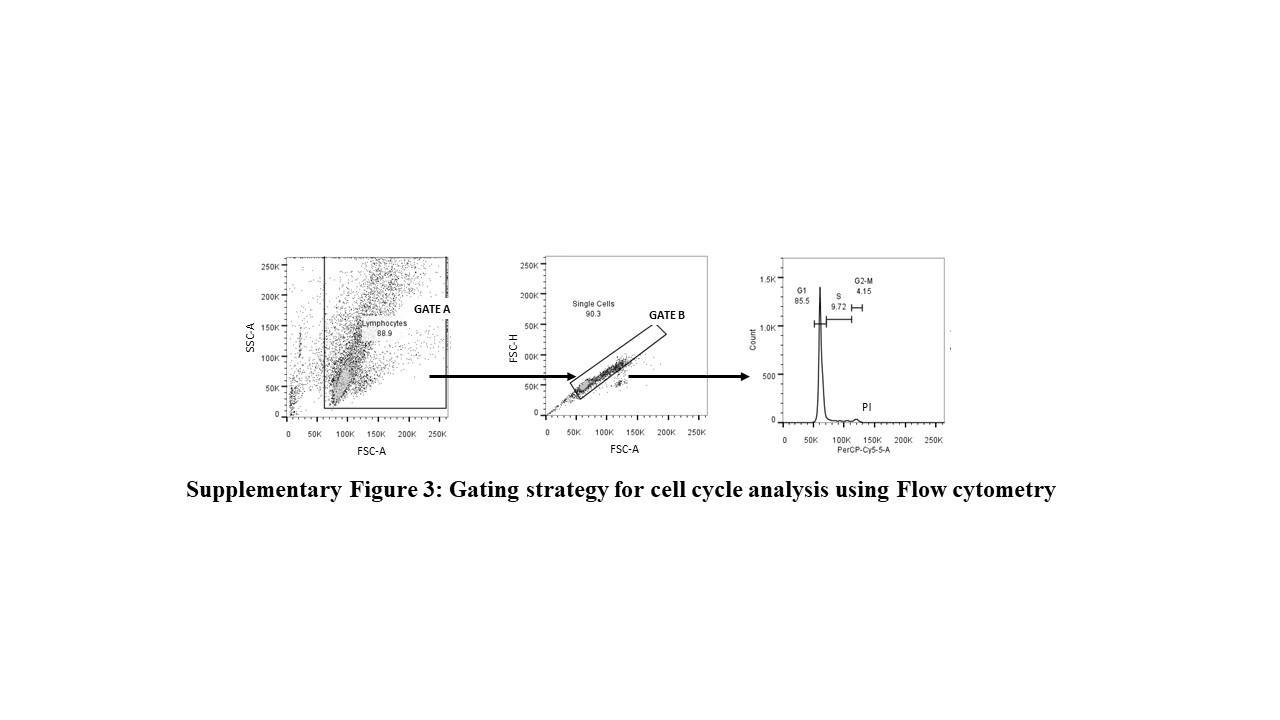

Supplement: Supplementary file 3 — Supporting information [file IID3-8-568-s003.jpg]

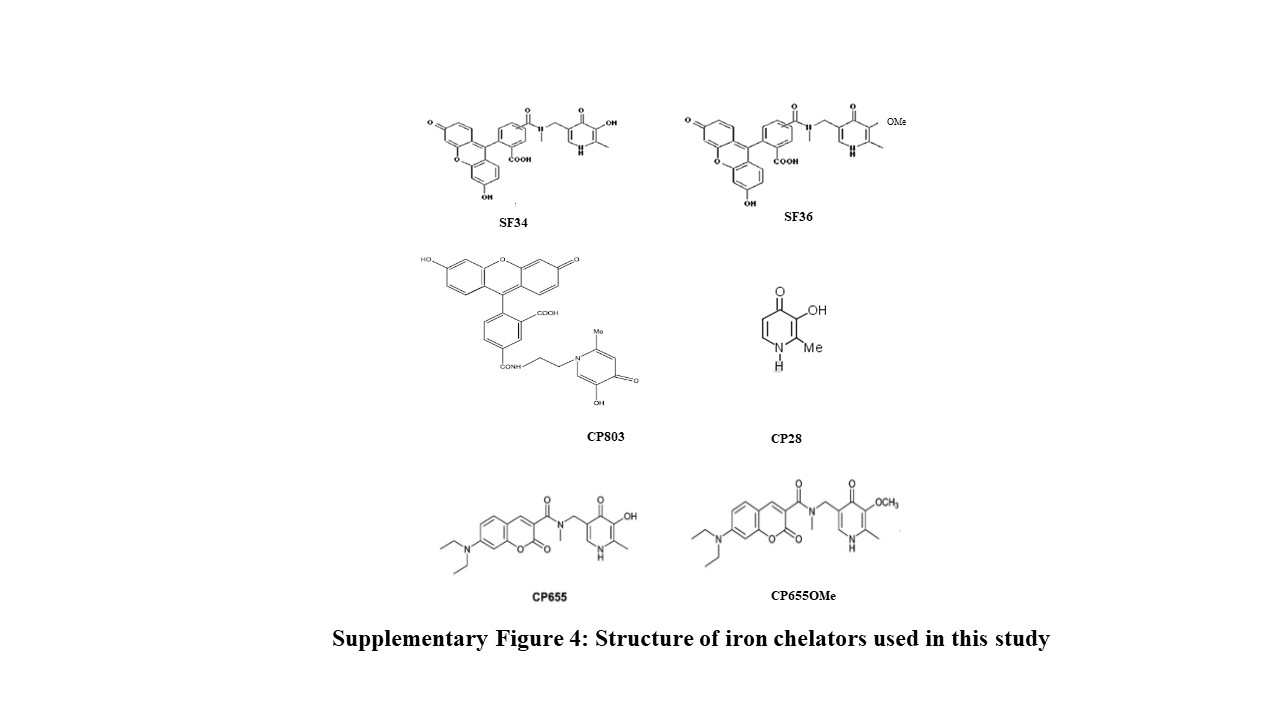

Supplement: Supplementary file 4 — Supporting information [file IID3-8-568-s004.jpg]
