# Supplementary material for: HPO iron chelator, CP655, causes the G1/S phase cell cycle block via p21 upregulation
Source: Immun Inflamm Dis. 2020 Aug 31;8(4):568–83. doi: 10.1002/iid3.342 (PMC7654408; doi:10.1002/iid3.342)
Supplement: Supplementary file 5 — Supporting information [file IID3-8-568-s005.docx]

**Supplementary Figure Legends**

**Supplementary Figure 1:** **Effect of CP655 on human T cell proliferation and cytokine production.** CD4+ T cells and CD14+ monocytes were isolated from fresh blood of healthy donors. Cells were mixed in a ratio of 2:1, stimulated with Tetanus Toxoid and treated with or without chelators. After 6 days of incubation, cells were stimulated with 750ng/ml Ionomycin and 50ng/ml PMA for four hours. Proliferation was measured by incorporation of ^3^H thymidine (A). Cytokines were measure by ELISA (B, C). Results from individual donors tested with different chelators shown as a percentage of cells from untreated cultures. Each symbol represents individual donor. Dotted line represents untreated control cells. Horizontal bar in A, B and C indicates the Median value. n=7-8 individual donors.

**Supplementary Figure 2: Kinetics of effect of CP655 on CD4+ T cells.** CD4+ T cells were isolated from blood of healthy donors. Cells were stimulated with anti-CD3/CD28 beads in a ratio of 1:20 in the presence of either of 5µM CP655 or 5µM CP655OMe from 4 hours – 48 hours. At each time point proliferation was measured by incorporation of ^3^H thymidine. **p<0.01, * p<0.05 calculated using paired t-test. Data represented as mean +SEM from n=3 individual donors.

**Supplementary Figure 3: Gating strategy for cell cycle analysis using Flow cytometry.** CD4+ T cells were isolated from fresh PBMCs of healthy donors. Cells were stimulated with 1 : 5 bead:cells ratio of anti-CD3/CD28 beads in the presence or absence of either CP655 (5µM) or CP655OMe (5µM) for 48 hours. Live lymphocytes were gated based on forward and side scatter (Gate A). Single cell population (Gate B) of Propidium Iodide stained cells were gated for height and area closest to 1. This data from gate B was acquired as a Histogram on a linear scale where the x-axis represented the DNA content and the y-axis showed the relative cell number. Analysis was conducted using the FlowJo software and the histogram was divided into G0/G1, S and G2/M phase based on the amount of DNA content.

**Supplementary Figure 4: Structure of iron chelators used in this study** (Ma *et al*., 2006; Fakih *et al*., 2009)
